# Recent progress on the role of non-coding RNA in postoperative cognitive dysfunction

**DOI:** 10.3389/fncel.2022.1024475

**Published:** 2022-10-13

**Authors:** Yu-Shen Yang, Shi-Ling He, Wei-Can Chen, Cong-Mei Wang, Qiao-Mei Huang, Yan-Chuan Shi, Shu Lin, He-fan He

**Affiliations:** ^1^Department of Anesthesiology, The Second Affiliated Hospital of Fujian Medical University, Quanzhou, China; ^2^Neuroendocrinology Group, Garvan Institute of Medical Research, Sydney, NSW, Australia; ^3^Faculty of Medicine, UNSW Sydney, Sydney, NSW, Australia; ^4^Centre of Neurological and Metabolic Research, The Second Affiliated Hospital of Fujian Medical University, Quanzhou, China

**Keywords:** postoperative cognitive dysfunction (POCD), non-coding RNA (ncRNA), microRNA, long non-coding RNA (lncRNA), circular RNA (circRNA)

## Abstract

Postoperative cognitive dysfunction (POCD), especially in elderly patients, is a serious complication characterized by impairment of cognitive and sensory modalities after surgery. The pathogenesis of POCD mainly includes neuroinflammation, neuronal apoptosis, oxidative stress, accumulation of Aβ, and tau hyperphosphorylation; however, the exact mechanism remains unclear. Non-coding RNA (ncRNA) may play an important role in POCD. Some evidence suggests that microRNA, long ncRNA, and circular RNA can regulate POCD-related processes, making them promising biomarkers in POCD diagnosis, treatment, and prognosis. This article reviews the crosstalk between ncRNAs and POCD, and systematically discusses the role of ncRNAs in the pathogenesis and diagnosis of POCD. Additionally, we explored the possible mechanisms of ncRNA-associated POCD, providing new knowledge for developing ncRNA-based treatments for POCD.

## Introduction

Postoperative cognitive dysfunction (POCD) is a common complication that occurs mainly in older adults undergoing surgery. It is characterized by impaired thought, memory, orientation, and executive abilities after anesthesia and surgery. Some evidence suggests that POCD increases morbidity and mortality rates, as well as hospitalization duration and costs (Steinmetz et al., 2009). Whilst the neuropathogenesis of POCD remains to be fully understood, it has been reported that neuroinflammation, neuronal apoptosis, oxidative stress, Aβ accumulation, and tau hyperphosphorylation are important in the pathogenesis.

Recently, accumulating proofs have emphasized a remarkable role for non-coding RNAs (ncRNAs) in the pathophysiology of POCD (Wang et al., 2021a; Wei et al., 2021a; Mao et al., 2022). ncRNAs are a set of RNA sequences that cannot be translated into proteins. This non-coding sequence involves various ncRNA families (such as microRNAs (miRNAs), long non-coding RNAs (lncRNAs), circular RNAs (circRNAs), and PIWI-interacting RNAs, etc.), the majority of which modulate epigenetic processes. Given that ncRNAs play critical roles in mediating post-transcriptional regulation of gene expression, their dysregulation may be at the core of the molecular mechanisms of POCD.

This review aims to discuss the evidence of ncRNAs involvement in POCD pathogenesis, diagnosis, and prognosis, including potential mechanisms, which may be used to develop novel therapeutics.

## Postoperative cognitive dysfunction

### Current views on postoperative cognitive dysfunction: Risk factors, definition, and diagnosis

Since the first official report of POCD was published in 1955, subsequent research on POCD has focused on different aspects of the disease, including its epidemiology, etiologies, diagnosis and treatment (Bedford, 1955). The risk factors of POCD are multifactorial, mainly involving the following five aspects: (1) patient factors such as age, sex, education level, history of preoperative cognitive impairment, physiological disturbances, and genetic factors; (2) anesthesia factors such as anesthesia approaches, duration of anesthesia, anesthetic types; (3) surgery factors such as surgery type and operation time; (4) intraoperative and postoperative complications such as blood loss during surgery, postoperative pain, infections, and respiratory complications; (5) environmental factors such as noise, bright, and light. Of these risk factors, advanced age has been demonstrated to be an independent risk factor for POCD both in clinical and animal studies. Reportedly, the ratio of aged patients (> 60 years of age) with cognitive impairment at three months after surgery was twice that of young and middle-aged people (12.7% vs. 5.7% vs. 5.6%) (Monk et al., 2008; Silbert et al., 2014). Alarmingly, the 1-year mortality for patients with POCD within three months after surgery was reported to be almost twice as high as that of patients without POCD (Fodale et al., 2010). Thus, the management of postoperative cognitive function is crucial for patients with these high-risk factors, especially for elderly patients.

Accurate diagnosis is prerequisite for treatment. According to the recommendation of the International Perioperative Cognition Nomenclature Working Group in 2018, POCD is defined as a cognitive impairment occurring from 30 days to 12 months after surgery, which cannot be interpreted by any other medical condition (Evered et al., 2018a). This multi-professional working group recommended the use of the Diagnostic and Statistical Manual of Mental Disorders fifth edition (DSM-5) to make the above diagnoses. The DSM-5 lists six core elements that should be considered when applying the diagnostic criteria for neurocognitive impairment: learning and memory, perceptual-motor function, language, complex attention, social cognition, and executive function. Subsequently, Butz and co-workers proposed another widespread criterion for POCD in 2019, namely a decline of one standard deviation from before to 3 months after surgery in at least two objectively measured cognitive functions (including cognitive flexibility, attention, verbal memory, visuomotor abilities, and language) (Butz et al., 2019).

Although the 2018 and 2019 proposals provide a formal definition of POCD, a uniform diagnostic criterion is still missing. In fact, the diagnostic methods of POCD are varied and can be summed up in changes in behavioral and pathological biomarkers. The specific strategies for POCD diagnosis in clinical practice and basic research exist some overlaps and are presented in Table 1. The treatment of POCD benefits from the improvement of diagnosis methods. However, plasma measurements of acute inflammatory markers (S-100β, tumor necrosis factor (TNF)-α, and interleukin (IL)-6) during the early postoperative period cannot precisely predicate the postoperative neurocognitive function, especially for late POCD (Sahoo et al., 2019). Thus, an in-depth understanding of the molecular mechanism of POCD is required to provide a set of carefully selected biomarkers with adequate specificity and sensitivity for POCD.

**TABLE 1:** The frequently used diagnosis methods for POCD.

	Clinical settings	References	Basic experiment	References		Testing content	Indicators	References
*Neuropsychological tests (which are different in clinical settings and basic experiment)*	Mini-Mental State Examination	May et al., 2022	Novel object recognition test	Rajagopal et al., 2014	*Pathological and molecular examination (which are universal in clinical settings and basic experiment)*	Brain-derived biomarkers	Aβ, Tau, S100β, NSE, BDNF, KYNA, GFAP, NSP	Szwed et al., 2021; Ji and Li, 2022; Wiberg et al., 2022
	Montreal Cognitive Assessment	Hobson, 2015	Y-maze test	Prieur and Jadavji, 2019		Inflammation-related biomarkers	TNF-α, IL-1, IL-6, IL-10, CRP	Fu et al., 2022
	Wechsler Memory Scale	Choi et al., 2014	Morris water maze test	Salman et al., 2019		Neurotransmitter-based biomarkers	IGF-1, AChE	Schaefer et al., 2019; Majewski et al., 2020
	Trail Making Test	Arbuthnott and Frank, 2000	Open field test	Knight et al., 2021		ncRNA-based biomarkers	miRNA-21-5p, circR-089763, miR-155	Wu et al., 2016; Wang et al., 2019; Szwed et al., 2021
	Rey Auditory Verbal Learning Test	Moradi et al., 2017	Contextual fear-conditioning test	Malikowska-Racia et al., 2018		Pathological examination of brain tissues	–	–
	Hopkins Verbal Learning Test	González-Palau et al., 2013						

Aβ, β-amyloid; AChE, acetylcholinesterase; BDNF, brain-derived neurotrophic factor; CRP, C-reactive protein; GFAP, glial fibrillary acidic protein; IGF-1, insulin growth factor-1; IL, interleukin; KYNA, kynurenic acid; NSE, neuron-specific enolase; NSP, neuroserpin; S100β, calcium-binding protein; Tau, a microtubule-stabilizing protein; TNF-α, tumor necrosis factor-α.

### Known cellular and molecular mechanisms of postoperative cognitive dysfunction

#### Neuroinflammation

Neuroinflammation exerts a vital impact on the initiation and development of POCD (Subramaniyan and Terrando, 2019). Excessive release of peripheral cytokines caused by anesthesia/surgery can disturb the blood-brain barrier (BBB) and activates microglia, thus triggering neuroinflammation and cognitive dysfunction (Hovens et al., 2012). The activated microglia continues to produce pathological cytokines, such as CD11b, IL-1β, and brain derived neurotrophic factor creating a vicious cycle of neuroinflammation (Qiu et al., 2016). Additionally, activated hippocampal microglia induced by etomidate administration triggers a neurotoxic-specific astrocyte response and causes long-term synaptic inhibition and cognitive dysfunction (Li et al., 2020a). Thus, microglial activation may be the key feature of POCD. Intriguingly, however, microglial activation does not always occur post-surgery. Some authors recently reported that glial activity significantly decreased 3–4 days after prostatectomy (Forsberg et al., 2017). This finding suggests that microglia may be involved in the interaction between neuroinflammation and POCD, and other factors may also be involved in this process.

#### Neuronal apoptosis

Surgery/anesthetics may induce neuronal apoptosis, leading to cognitive impairment (Mao et al., 2022). This process does not appear to be related to neuroinflammation or Aβ accumulation. However, it is potentiated by these pathological changes in CNS. Surgery/anesthetics can trigger the activation of NF-κB-mediated inflammation pathway in neurons, which in turn induce neuronal apoptosis (Yuan et al., 2022). Anesthetics also activate the apoptosis-related protein caspase-1, which induces neuroinflammation (Shao et al., 2020). This bidirectional communication also exists between neuronal apoptosis and changes in Aβ peptide (Xie et al., 2007). Therefore, these mechanisms may create a self-perpetuating cycle, in which surgery/anesthetics induces apoptosis that in turn worsens neuroinflammation and Aβ aggregation. These changes accelerate apoptosis, ultimately resulting in cognitive decline.

#### Oxidative stress

In response to surgery/anesthetics, a large amount of reactive oxygen species is produced due to the overproduction of free radicals and damage to the antioxidant defense system. Reactive oxygen species can modify nucleic acids, lipids, and proteins in the CNS, and have been implicated in neuroinflammation, Aβ deposition, tau protein hyperphosphorylation, and mitochondrial dysfunction (Reynolds et al., 2007). These reactive oxygen species generated during oxidative stress damage vulnerable neurons, contributing to cognitive dysfunction. In a rodent model of POCD, exploratory laparotomy with isoflurane anesthesia increased the expression of Nox2 (a predominant source of reactive oxygen species) in the brain and impaired the contextual fear memory at 7 days post-surgery, indicating activated oxidative stress in the hippocampus may be associated with the pathogenetic mechanism of POCD (Qiu et al., 2016).

#### Aβ deposition and tau hyperphosphorylation

Aβ deposition and tau phosphorylation are recognized mechanisms for the pathogenesis of Alzheimer’s disease. Excessive Aβ deposition and tau hyperphosphorylation activates the pathogenetic cascade that results in the disruption of the surrounding neuron and synapse structure, signaling dysfunction, intracellular neurofibrillary tangles, and cognitive impairment (Salminen et al., 2013; Song et al., 2014). There is increasing evidence for these proteins role in POCD. In a clinical trial with the aim to investigate the association between POCD and plasmatic Aβ1–42 concentrations, Požgain et al. (2022) demonstrated the earlier suspected relationship between plasmatic Aβ1–42 concentration and POCD, implicating Aβ proteins in the pathogenesis of early POCD (Požgain et al., 2022). Furthermore, a pre-clinical study showed that isoflurane exposure increased the levels of Aβ and phosphorylated tau protein in brain, and lead to the impairment of spatial memory, probably through the activation of the A1A receptor (Zuo et al., 2018). These findings indicate that POCD might share a common pathogenic mechanism with Alzheimer’s disease.

#### Brief summary

**FIGURE 1:**
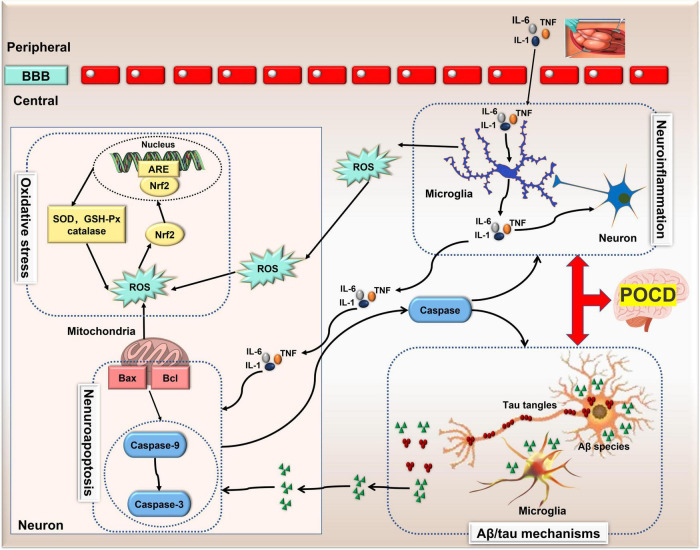
The pathological mechanisms of POCD. Neuroinflammation, Aβ deposition/tau hyperphosphorylation, neuronal apoptosis and oxidative stress are four recognized pathological mechanisms involved in POCD. Neuroinflammation can induce neuronal apoptosis and oxidative stress via microglia-secreted cytokines and reactive oxygen species, and in turn, apoptosis-related protein caspase-1 exacerbates neuro-inflammation. However, this bidirectional communication also exists between neuronal apoptosis and changes in Aβ peptide. Thus, these mechanisms can interact with one another exacerbating the pathological outcomes of POCD. Aβ, amylase-β; ARE, antioxidant response element; BBB, blood brain barrier; GSH, glutathione; IL, interleukin; lncRNA, long non-coding RNA; nuclear factor-erythroid 2-related factor 2 (Nrf2); ncRNA, non-coding RNA; POCD, postoperative cognitive dysfunction; ROS, reactive oxygen species; SOD, superoxide dismutase; TNF, tumor necrosis factor.

The specific mechanism of POCD is still unknown, but several hypotheses, including neuroinflammation, neuroapoptosis, oxidative stress and Aβ deposition/tau hyperphosphorylation have been demonstrated and recognized. Remarkably, these mechanisms can interact with one another to exacerbate the pathological outcomes of POCD (Figure 1). A better understanding of the molecular mechanisms behind this interaction may facilitate the development of therapy for POCD. Recently, a growing body of evidence implicates the importance of ncRNAs in the onset and development of POCD.

### The role of ncRNAs in postoperative cognitive dysfunction

**FIGURE 2:**
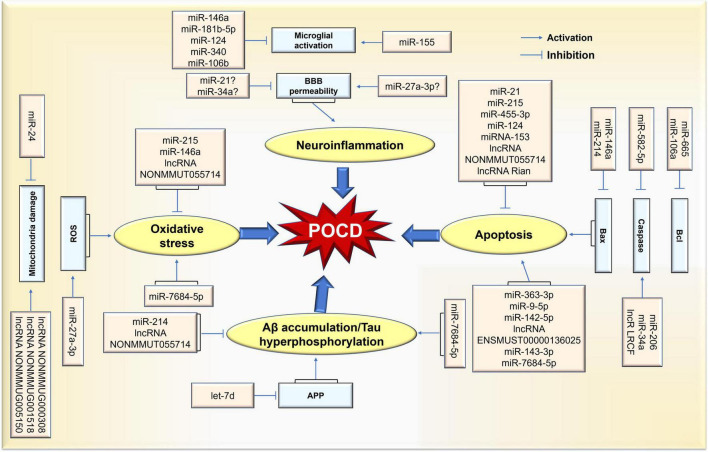
Schematic representation of ncRNA-mediated pathological process in POCD. Differential expression of ncRNAs caused by surgical/anesthesia results in various functional changes in brain via coordination of network(s) where key targets are upregulated or downregulated. Thus, POCD-associated pathological changes are evident in neuroinflammation, neuronal apoptosis, and oxidative stress rates, as well as Aβ accumulation/tau hyperphosphorylation.

Among the ncRNAs in the field of cognitive function, the miRNA, lncRNA and circRNA families are the most valuable clinically classes due to their powerful gene regulation capabilities. miRNAs are about 22–25 nucleotides in length and have the functions of RNA silencing and post-transcriptional regulation of gene expression. lncRNAs are more than 200 nucleotides in length and are involved in a variety of epigenetic modulations. circRNAs are a new class of transcripts characterized by covalently linked 5′ and 3′ ends that modulate the stability or translation efficiency of target mRNAs. In recent years, the research progress of ncRNAs has completely changed our understanding of POCD-related molecular mechanisms. It has been demonstrated that ncRNAs play vital role in several major pathological processes of POCD, including neuroinflammation, Aβ accumulation/tau hyperphosphorylation, neuronal apoptosis, and oxidative stress. Meanwhile, the diagnostic value of ncRNAs in POCD has been increasingly revealed. Thus, this review will elucidate the role of ncRNAs in POCD in the following section (Figure 2).

#### The diagnostic value of ncRNAs in postoperative cognitive dysfunction

Anesthetic agents and surgical trauma may affect gene expression in the CNS (Whitaker et al., 2019); detecting changes in ncRNAs expression may support early diagnosis of POCD. In contrast to peripheral tissues with easy access, brain tissue is difficult to obtain from live patients. However, when brain diseases occur, ncRNA-bearing exosomes, extracellular vesicles with diameter of less than 150 nm, are released, cross the BBB, and enter the blood circulation (Wang et al., 2022b); these circulating ncRNAs carry the information from the brain and can be used as a biomarker of POCD that can be detected by non-invasive methods outside of the brain. Encouragingly, some promising results regarding the clinical significance of circulating ncRNAs [such as miR-155 (Wu et al., 2016), circ-089763 (Wang et al., 2019; Zhou et al., 2020), miR-21-5p (Szwed et al., 2021)] in POCD have been obtained recently.

#### ncRNAs and neuroinflammation in postoperative cognitive dysfunction

Activation of inflammation-related cells and changes in cytokine levels caused by tissue damage are closely associated with abnormal expression levels of ncRNAs. ncRNAs may either restrict or facilitate neuroinflammation by intervening in major pathological processes of neuroinflammation, such as blood brain barrier disruption and microglial activation, thereby ameliorating or aggravating the pathological outcome of excessive inflammatory responses.

The damage to the BBB is implicated in many neuroinflammatory disorders including POCD (He et al., 2012). A growing body of evidence suggests that brain-enriched ncRNAs affect the BBB function under physiological and pathological conditions (Zuo et al., 2019). For example, in an *in vitro* model of the brain endothelium, miR-27a-3p was demonstrated to strengthen the intercellular junctions of the brain endothelium and control the permeability of the endothelial barrier (Harati et al., 2022). However, in rodent models of encephalopathy, miR-21 and miR-34a negatively affected the permeability of the BBB, enhancing ischemia-induced leakage, by regulating tight junction proteins (Ma et al., 2020). While the association between ncRNAs and POCD has been documented (Twaroski et al., 2014; Li et al., 2018; Zhu and Ma, 2021), direct experimental evidence is lacking for the relationship between these ncRNA molecules and surgery/anesthesia-induced BBB disruption.

It is reported that ncRNAs may affect the development of POCD via the modulation of functional phenotype of microglia. In a rodent model of POCD, overexpression of miR-146a abrogated hippocampal-dependent learning and memory impairment, accompanied by decreased expression of downstream targets of miR-146a (IRAK1 and TRAF6) and the suppression of the hippocampal microglia activity (Chen et al., 2019a). The downregulated ncRNA miR-181b-5p in the hippocampus of POCD mice inhibited the expression of its target TNF-α by binding to the 3’-untranslated region (UTR) of TNF-α and reduced the activation of the microglia (Lu et al., 2019). miR-124 is abundant and affects microglial activity in the brain. In a recent *in vitro* model, miR-124 overexpression with a concomitant decrease of its target (VAMP3) expression led to a decrease of inflammatory factors associated with microglial activation post-surgery (Chen et al., 2019b). Other microRNA types and their targets, including miR-155/SOCS1 (Zheng et al., 2018), and miR-106b/Dusp9 (Liu et al., 2021), were suggested to regulate microglial function in LPS-activated microglia treated by anesthetic agents.

#### ncRNAs and neuronal apoptosis in postoperative cognitive dysfunction

Apoptosis is a type of programmed cell death. Bax and Bak are Bcl-2 family members that mediate cell apoptosis. Caspases play critical roles in regulating apoptosis. In particular, caspase-3 can catalyze the cleavage of pivotal cellular elements, participating in brain maturation, and DNA disruption and chromatin aggregation during apoptosis. Studies have shown that surgery/anesthesia-induced neuronal apoptosis plays a crucial role in the occurrence and development of POCD, and its mechanism is related to ncRNAs.

In a mouse model of POCD, Mao and colleagues demonstrated that the elevation of miR-146a levels could target BTG2 expression and suppressed neuronal apoptosis, thus ameliorating POCD following probiotics (De Simone Formulation) treatment (Mao et al., 2022). miR-214, an upstream target of Bax, has been implicated in neuronal apoptosis induced by various anesthetics, such as isoflurane (Yan et al., 2013), desflurane (Yu and Zhang, 2013), and propofol (Guo et al., 2018). Historically, miR-665 in anesthesia-induced neurotoxicity was believed to directly down-regulate anti-apoptotic gene *Bcl-2l1* expression, increasing caspase-3-mediated apoptosis rates (Sun and Pei, 2015, 2016; Sun et al., 2015; Jiang et al., 2019). Recently, an increasing number of microRNA molecules [miR-21 (Twaroski et al., 2014), miR-206 (Li et al., 2017), miR-34a (Li et al., 2018), miR-215 (Tang et al., 2020), miR-363-3p (Yao and Zhang, 2020), miR-455-3p (Zhu et al., 2020), miR-9-5p (Zhang et al., 2022), and miR-582-5p (Zhang et al., 2020b)] have been revealed to be associated with propofol-induced neuronal apoptosis. Meanwhile, the relationship between several microRNA types [miR-124 (Yang et al., 2019), miR-153 (Shao et al., 2019), miR-106a (Zhang et al., 2020a), and miR-142-5p (Xie et al., 2020)] and isoflurane-induced neuronal apoptosis has been demonstrated.

Chen et al. (2016) found that 31 lncRNA molecules were significantly dysregulated (19 upregulated and 12 downregulated) in the hippocampus of sevoflurane-treated neonatal mice. These authors further reported that the upregulation of lncENSMUST00000136025, one of the dysregulated lncRNA types, most likely resulted in the overexpression of *Bcl-211*, which promoted mitochondrial-mediated apoptosis (Chen et al., 2016). Subsequently, other lncRNA types, such as NONMMUT055714 (Wei et al., 2021a), Rian (Yu et al., 2021), and LRCF (Zeng et al., 2021), have been indicated to be implicated in anesthesia-mediated neuroapoptosis.

#### ncRNAs and oxidative stress

Imbalance between cellular reactive oxygen species level and antioxidant defense activity leads to excessive oxidative stress after surgery/anesthesia, which is toxic to neurons. Lipid peroxidation may cause cell disruption and death. Therefore, oxidative stress is key to neuronal injury and POCD. Recent studies have indicated that surgery/anesthesia-induced oxidative stress is strongly linked to ncRNAs.

Peroxisome proliferator-activated receptor γ (PPARγ) is one of the targets of miR-27a-3p. Upregulation of miR-27a-3p expression can deteriorate sevoflurane-induced neuron apoptosis via increasing oxidative stress-related protein expression through the suppression of PPARγ (Lv et al., 2017). In isoflurane-treated rat hippocampal neurons, Li et al. (2020b) demonstrated that miR-24 overexpression suppressed oxidative damage, mitochondrial size, and Ca^2+^ permeability in hippocampal neurons by targeting p27*^kip^*^1^ (Li et al., 2020b). A recent study explored the mechanisms of microRNAs regulation of propofol exposure-mediated neurotoxicity *in vitro*. The results suggest that miR-215 mimics can inhibit oxidative stress by inhibiting reactive oxygen species production and malondialdehyde and lactate dehydrogenase secretion, and increasing superoxide dismutase levels by targeting large tumor suppressor 2 (LATS2) (Tang et al., 2020).

In addition, three lncRNA types have been identified as differentially expressed molecules and have been associated with a range of pathological processes such as mitochondrial dysfunction, oxidative stress, aging-related metabolic alterations, DNA damage, and apoptosis, as well as neurodegenerative features during sevoflurane anesthesia (Qu et al., 2020). Furthermore, in a POCD mouse model, other lncRNA types, such as NONMMUT0557143, regulated the expression of oxidative stress-related markers (Wei et al., 2021a), such as malondialdehyde.

#### ncRNAs and excessive Aβ deposition/tau hyperphosphorylation in postoperative cognitive dysfunction

miR-214 is involved in anesthesia-induced Aβ accumulation and cell death by binding to Bax 3’UTR (Yan et al., 2013; Yu and Zhang, 2013), and several studies have reported a close relationship between ncRNAs and Aβ deposition/tau hyperphosphorylation in POCD. Luo et al. (2015) demonstrated that downregulation of miR-let-7d can lead to isoflurane-induced impairment of learning and memory by directly up-regulating its target amyloid precursor protein, thereby increasing Aβ production. In addition, Wei et al. (2021a) also indicated that lnc NONMMUT055714 downregulation is associated with increased Aβ and p-tau expression via competing endogenous RNA (ceRNA)-mediated regulation of miR-7684-5p expression in isoflurane/orthopedic surgery-induced POCD model.

#### Brief summary

The evidence supports the notion that ncRNAs are highly important in POCD-related pathological processes and promising targets for the treatment of POCD. Furthermore, several important phenomena were found by reviewing the ncRNA expression alteration in *in vitro* and *in vivo* models induced by anesthetics and/or surgery (Table 2). First, the ncRNAs expression profile differs depending on the anesthetics/surgery regimen. For example, miR-214 downregulation was observed in POCD rat models induced by isoflurane and desflurane and subsequent overexpression improved cognitive function (Yan et al., 2013; Yu and Zhang, 2013). Thus, miR-214 overexpression could be regarded as a protective factor for postoperative cognitive function. Contrastingly, in propofol-induced POCD rats, miR-214 expression was significantly upregulated (Guo et al., 2018). Hence, we speculate that ncRNA expression patterns and biological functions vary according to individual differences and anesthetic procedures.

**TABLE 2:** The anesthetics and/or surgery-specific ncRNAs alterations and their targets in POCD.

Drug types	Anesthetics	ncRNA alterations	Known target(s)	Animal	Interventions	Function	References
Inhaled anesthetics	Isoflurane	miR-146a↑	IRAK1, TRAF6	Male mouse	Orthopedic surgery	NI	Chen et al., 2019a
		miR-181b-5p↓	TNF-α	Male mouse	Orthopedic surgery	NI	Lu et al., 2019
		miR-7684-5p↓	SORLA	Male mouse	Orthopedic surgery	NI, NA, OS, A/T	Wei et al., 2021a
		miR-153↓	Nrf2	Male mouse	Inhalation anesthesia	NA, OS	Shao et al., 2019
		miR-106a↑	LIMK1	Male mouse	Inhalation anesthesia	NA	Zhang et al., 2020a
		miR-let-7d↓	APP	Male rats	Inhalation anesthesia	A/T	Luo et al., 2015
		miR-203↑	−	Male rats	Inhalation anesthesia	−	Cao et al., 2012
		miR-214↓	Bax	Male rats	Inhalation anesthesia	NA, A/T	Yan et al., 2013
		miR-124↓	EGR1	Rats*	Inhalation anesthesia	NA	Yang et al., 2019
		miR-24↓	p27^kip1^	Rats*	Inhalation anesthesia	NA, OS	Li et al., 2020b
		miR-142-5p↑	−	Rats*	Inhalation anesthesia	NA	Xie et al., 2020
		lnc NON-MMUT055714↓	miR-7684-5p	Male mouse	Orthopedic surgery	NI, NA, OS, A/T	Wei et al., 2021a
		lnc H19↑	miR-17-5p	*In vitro* model		NI	Ge et al., 2021
		lnc MEG3↑	miR-7-5p	*In vitro* model		NI, NA, OS	Li et al., 2022a
	Sevoflurane	miR-27b-3p↓	Hoxa5	Male mouse	Inhalation anesthesia	NI	Zhu and Ma, 2021
		miR-27a-3p↑	PPARγ	Male mouse	Inhalation anesthesia	NI, NA, OS	Lv et al., 2017
		miR-410-3p↓	CXCR5	Male rats	Inhalation anesthesia	NI, NA	Su et al., 2019
		miR-424↓	TLR4	Male rats	Inhalation anesthesia	NI, NA	Li et al., 2022b
		miR-27b↑	LIMK1	Male rats	Inhalation anesthesia	−	Yu et al., 2016
		miR-329-3p↓	E2F1, NF-κB P65	Male rats	Inhalation anesthesia	NI	Chen et al., 2022
		miR-124↓	Capn4	*In vitro* model		NI, NA	Zhao et al., 2021
		lnc ENSMUST00000 136025↑	Bim	Male mouse	Inhalation anesthesia	NA	Chen et al., 2016
		lnc Rian↓	miR-143-3p	Mouse*	Inhalation anesthesia	NA, OS	Yu et al., 2021
		lnc Rik-203↓	miR-101a-3p	Mouse*	Inhalation anesthesia	−	Zhang et al., 2019
	Desflurane	miR-214↓	Bax	Male rats	Inhalation anesthesia	NA, A/T	Yu and Zhang, 2013
Intravenous anesthetics	Propofol	miR-34a↑	MEK1	Male rats	Anesthesia	NA	Li et al., 2018
		miR-214↑	PTEN, NF-κB	Rats*	Anesthesia	NA	Guo et al., 2018
		miR-155↓	SOCS1	*In vitro* model		NI	Zheng et al., 2018
		miR-106b↑	Dusp9	*In vitro* model		NI	Liu et al., 2021
		miR-21↓	Sprouty 2	*In vitro* model		NA	Twaroski et al., 2014
		miR-665↑	Bcl-2l1	*In vitro* model		NA	Sun and Pei, 2015
		miR-206↑	PUMA	*In vitro* model		NA	Li et al., 2017
		miR-215↓	LATS2	*In vitro* model		NA, OS	Tang et al., 2020
		miR-363-3p↑	CREB	*In vitro* model		NA, OS	Yao and Zhang, 2020
		miR-455-3p↓	EphA4	*In vitro* model		NA	Zhu et al., 2020
		miR-9-5p↑	CXCR4	*In vitro* model		NA	Zhang et al., 2022
		miR-582-5p↓	ROCK1	*In vitro* model		NA	Zhang et al., 2020b
		lncRNA-LRCF↑	miR138-5p	*In vitro* model		NA	Zeng et al., 2021
		circR 001372↓	miR-148b-3p	Rats*	Anesthesia	NI, NA	Wang et al., 2021a
Others	Pentobarbital sodium	miR-146a↓	BTG2	Male mouse	Splenectomy	NA, OS	Mao et al., 2021
		miR-124↓	VAMP3	Male rats	Laparotomy	NI	Chen et al., 2019b

*Information about the sex of experimental animal was not provided by authors. A/T, Aβ deposition and tau hyperphosphorylation; NA, neuronal apoptosis; NI, neuroinflammation; OS, oxidative stress.

An additional concern is the predominant use of males to establish the POCD model. In fact, sex differences during development cause altered responses to anesthetics (Gan et al., 1999; Xie et al., 2015). Recently, Schenning et al. (2019) demonstrated sex and genetic differences in POCD in a longitudinal study. The study demonstrated that men with an APOE4 allele performed significantly worse than women APOE4 carriers on cognitive testing following surgery and anesthesia (Schenning et al., 2019). Similarly, ncRNA expression patterns show sex differences in brain after anesthetics exposure. In neonatal piglets anesthetized with 2% isoflurane, Whitaker et al. (2019) found no overlap in miRNA expression in hippocampus tissue between isoflurane-exposed males and females, suggesting sex differences in isoflurane-induced miRNA expression (Whitaker et al., 2019). Thus, male-biased results of ncRNAs expression in POCD may not be applicable to females. More studies in female models are required to examine the role of ncRNAs in POCD etiology.

In addition to sex, onset time of postoperative cognitive change might alter ncRNAs expression profile. According to the latest consensus (Evered et al., 2018b), the term of “perioperative neurocognitive dysfunction (PND),” further classified into postoperative delirium (POD, the early stage of postoperative cognitive changes occurred up to seven days after surgery) and POCD (the late stage of postoperative cognitive changes diagnosed thereafter until 12 months), is recommended to describe the overall cognitive function demonstrated during the perioperative periods. Evidence for the role of ncRNAs in POD has increased in the past few years (Ran et al., 2022; Wang et al., 2022a). However, in two different microarray studies, performed to identify differentially expressed lncRNAs and mRNAs in POD and POCD, no overlap was observed between POD and POCD patients (Zhang et al., 2018; Song et al., 2021). Thus, beyond the timing of diagnosis, ncRNAs expression pattern may become another distinguishing feature to separate POCD from POD.

### ncRNAS influence postoperative cognitive dysfunction through multiple signaling pathways

Researchers have reported multiple possible mechanisms by which ncRNAs contribute to POCD. As mentioned above, ncRNAs are extensively involved in the development of POCD. Next, we will discuss ncRNAs that may participate in the signaling pathways and molecular mechanisms of surgery/anesthesia induced cognitive impairment.

#### ceRNAs mechanism

Recently, ceRNA has been implicated in the physiology and development of various diseases. Coding RNA and ncRNAs (lncRNAs, circRNAs) molecules may compete for binding to miRNA through miRNA-responsive elements, thereby regulating miRNA-mediated gene silencing and expression. Some evidence suggests that the ceRNA regulatory network of lncRNA/circRNA-miRNA-mRNA contributes to POCD.

Wei et al. (2017) used bioinformatics methods to construct a ceRNA expression network and to identify the potential role of lncRNA as ceRNA in POCD. In this study, lncNONMMUT055714 bound competitively with miR-7684-5p, upregulating the expression of its target gene, *Sorl1* (Wei et al., 2017). In 2021, this academic team further demonstrated the protective effect of the ceRNA network of lncNONMMUT055714-miR-7684-5p on POCD (Wei et al., 2021a). Furthermore, Zhang et al. (2019) reported that lncRNA Rik-203 can promote neural differentiation by inhibiting the ability of miR-101a-3p to decrease GSK-3β levels. Importantly, two ceRNA networks [lncMEG3-miR-7-5p (Li et al., 2022a) and lncH19-miR-17-5p (Ge et al., 2021)] were revealed to regulate isoflurane-induced cognitive dysfunction. Recently, multiple teams have successfully constructed circRNA-miRNA-mRNA networks using bioinformatics methods, highlighting their role in POCD (Gao et al., 2020; Wu et al., 2021). Furthermore, the experimental evidence further demonstrated the effect of circ-001372-miR-148b-3p network on POCD (Wang et al., 2021a).

#### PI3K/Akt signaling pathway

Phosphoinositide 3-kinase (PI3K)/protein kinase B (Akt) signaling pathway plays a regulatory role in the occurrence and development of POCD. ncRNAs have neuromodulatory effects on POCD through the PI3K/Akt signaling pathway. In isoflurane-exposed rats or B35 neuron-like cells, miR-203 overexpression increased B35 cell tolerance to oxygen-glucose deprivation and phospho-Akt expression (Cao et al., 2012). While revealing the effect of miR-214 on propofol-induced neuronal apoptosis and the possible mechanism of its pro-apoptotic effect, Guo et al. (2018) found that miR-214 overexpression suppressed PI3K/Akt signaling by activating phosphatase and tensin homolog in the nerve cells treated with propofol (Guo et al., 2018). Subsequently, Wu and colleagues reported that miR-214 over-expression significantly inhibited isoflurane-induced reduction in the viability and inactivation of the PI3K/Akt pathway in SH-SY5Y cells (Wu et al., 2019). Furthermore, a recent study demonstrated that miR-410-3p could inhibit sevoflurane-induced apoptosis of the hippocampal neurons through the PI3K/Akt signaling pathway (Su et al., 2019). As a known regulator of miR-21, signal transducer and activator of transcription 3 plays a key role in propofol-induced neurotoxicity by mediating the miR-21/sprout 2/Akt pathway (Twaroski et al., 2014). Meanwhile, a recent RNA-sequencing analysis revealed that propofol-based sedatives may up-regulate FoxO pathway-related proteins (PI3K and Akt) and down-regulate FoxO3a at both the RNA and protein levels via differently expressed lncRNAs (lncE230001N04Rik, lncRP23-430H21.1, and lncB230206L02Rik) (Fan et al., 2018). In *in vitro* PC-12 cells and rat brain tissues, circ-001372 suppressed propofol-induced neurotoxicity and neuroinflammation through the PI3K/Akt signaling pathway (Wang et al., 2021a). This evidence suggest that ncRNAs-mediated PI3K/Akt signaling pathway plays an important role in anesthesia-induced cognitive impairment.

#### NF-κB signaling pathway

In POCD, nuclear factor kappa B (NF-κB) signal transduction is often upregulated and is associated with the dysregulation of ncRNAs expression. For example, miR-146a over-expression can restore POCD by inhibiting the NF-κB pathway via targeting IL-1 receptor-associated kinase (IRAK) and TNF receptor-associated factor 6 (TRAF6) in the hippocampus of mice (Chen et al., 2019a). In *in vitro* lipopolysaccharide (LPS)-stimulated BV2 cells, miR-340 mimics exert anti-inflammatory effects by inhibiting NF-κB pathways and proinflammatory cytokine activity (Bao et al., 2019). Recently, Li et al. (2022b) found that up-regulation of miR-424 attenuated sevoflurane-induced NF-κB phosphorylation, and up-regulation of *toll-like receptor 4 (TLR4)* and *myeloid differentiation factor 88 (MyD88)* was achieved by targeting the 3’-untranslated region of *TLR4* and inducing mRNA degradation (Li et al., 2022b). In addition, Zhao et al. (2021) found that miR-124 inhibited sevoflurane-induced apoptosis of mouse hippocampal neurons by targeting Capn4 and suppressing NF-κB signaling pathway (Zhao et al., 2021). In contrast, circ-001372 contributed to the improvement of anesthetics-related cognitive disorders by inhibiting the NF-κB signaling pathway (Wang et al., 2021a).

#### Other signaling pathway

LIM kinase 1 (LIMK1), a member of the LIM kinase family, regulates a variety of neurophysiological functions, including axon elongation, hippocampal synaptic plasticity, and long-term memory. Recent studies have shown that the downregulation of LIMK1 by miR-27b is associated with cognitive impairment in response to sevoflurane (Yu et al., 2016). Subsequently, miR-106a upregulation was found to decrease Bcl-2 levels, increase expression levels of Bax and cleaved caspase 3, and exacerbate isoflurane-induced cognitive impairment by targeting LIMK1 (Zhang et al., 2020a). In addition, lncRNA showed neuroprotective effects on sevoflurane-induced cognitive dysfunction by regulating the miR-143-3p/LIMK1 axis (Yu et al., 2021).

cAMP-response element binding protein (CREB) is a nuclear regulator in eukaryotic cells and contributes to learning, memory, synapse formation, and neuronal anti-apoptosis. In a rat model of cognitive dysfunction, miR-34a promoted propofol-induced expression of cleaved caspase-3/8 and Bax, which is accompanied by a significant increase in CREB (Li et al., 2018). Yao and Zhang (2020) reported that miR-363-3p binds to the 3’-UTR unit of the CREB transcript, inhibiting CREB activity, and promotes propofol-induced oxidative stress and apoptosis *in vitro* (Yao and Zhang, 2020). Several other signaling pathways involved in anesthesia-induced neurotoxicity have been described in recent years, including the miR-153/Nrf2/ARE (Shao et al., 2019), miR-455-3p/EphA4 (Zhu et al., 2020), miR-215/LATS2 (Tang et al., 2020), and miR-34a/MEK/ERK axes (Li et al., 2018).

#### Brief summary

**FIGURE 3:**
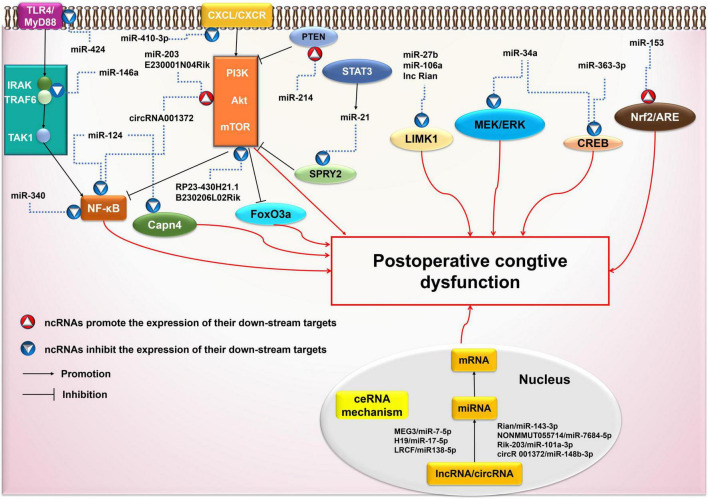
Possible ncRNAs-mediated signaling pathways in postoperative cognitive dysfunction (POCD). AKT, protein kinase B; ARE, antioxidant response element; circRNA: circular RNA; CREB, cAMP-response element binding protein; CXCR4, chemokine CXC receptor 4; ERK, extracellular signal-regulated kinase; FoxO3a, forkhead box O 3a; IRAK, IL-1 receptor-associated kinase; LIMK1, LIM kinase 1; lncRNA: long non-coding RNA; MAPK, mitogen activated protein kinase; miRNA: microRNA; mRNA: messenger RNA; mTOR, mammalian target of rapamycin; MyD88, myeloid differentiation factor 88; ncRNA: non-coding RNA; NF-κB, Nuclear Factor κB; Nrf2, nuclear factor-erythroid 2-related factor 2; PI3K, phosphatidylinositol 3 -kinase; SPRY2, Sprouty 2; STAT3, activated Signal Transducer and Activator of transcription 3; TAK1, TGFβ-activated kinase 1; TGF, transforming growth factor; TLR, toll-like receptor; TNF, tumor necrosis factor; TRAF6, TNF receptor-associated factor 6.

ncRNAs are involved in the activation of ceRNAs, PI3K/Akt, NF-κB, and other signaling pathways (Figure 3). The pathogenesis of cognitive impairment caused by surgical trauma/anesthesia may be related to the abnormal activation of these signaling pathways. Multiple pathways and mechanisms are involved in ncRNAs-associated POCD, creating many potential prevention targets. However, the specific downstream signaling cascades remain to be characterized, and further studies are required to identify them.

### Protective effects of ncRNAS-mediated dexmedetomidine on cognitive function

Dexmedetomidine, a highly selective alpha-2 adrenergic agonist, has analgesic, anxiolytic, and sedative effects, and is widely used as a perioperative sedative. Dexmedetomidine has been shown to have anti-inflammatory and neuroprotective effects, providing benefits in reducing the incidence of POCD (Liu et al., 2016). Although the exact mechanisms of its benefit remain to be determined, some evidence suggest that ncRNAs may contribute to the protective effect of dexmedetomidine on postoperative cognitive function.

In a lipopolysaccharide-induced rat neuroinflammation model, dexmedetomidine reduced the expression of two pro-inflammatory cytokines, *IL1-*β and *TNF-*α genes, in the hippocampus and cortex. Moreover, the lipopolysaccharide-mediated expressions of miR-124, 132, 134, and 155 were significantly reduced after dexmedetomidine exposure in both brain regions (Paeschke et al., 2017). In LPS-stimulated BV2 cells, miR-340 mimics enhanced the anti-inflammatory effects of dexmedetomidine by inhibiting NF-κB and pro-inflammatory cytokines such as IL-6, IL-1β, IL-2 (Bao et al., 2019). In addition, Wang et al. used C57BL/6J aging mice (18 weeks old) to establish a POCD model to determine the mechanisms of the protective effects of dexmedetomidine on POCD. These authors demonstrated that dexmedetomidine administration up-regulated miR-381 expression and downregulated the EGR1/p53 axis, thus protecting against cognitive impairment. These results suggest that dexmedetomidine exerts neuroprotective effects by activating the miR-381/EGR1/p53 pathway, thus providing benefits in preventing POCD in this model (Wang et al., 2021b). Two separate studies involving POCD mouse models induced by sevoflurane anesthesia revealed that dexmedetomidine alleviates sevoflurane-induced neurotoxicity via the miR-129/TLR4 axis (Wei et al., 2021c) and miR-330-3p/uncoordinated-51-like autophagy activating kinase 1 (ULK1) axis (Xu et al., 2021).

In another study, Deng et al. (2020) investigated the mechanism and function of dexmedetomidine-regulated lncRNA in improving POCD in rats. The results of lncRNA sequencing and quantitative PCR analyses in the dexmedetomidine group revealed a total of 60 differentially expressed lncRNA types (16 up-regulated and 44 downregulated), including lncLOC102546895, which may contribute to POCD by inhibiting the proliferation and promoting the apoptosis of microglia and the expression of the neuronal transcription factor Npas4 (Deng et al., 2020). In addition, circRNA molecules are involved in dexmedetomidine-mediated neuroprotection against POCD. In a POCD rat model, RNA sequencing revealed 164 differentially expressed circRNA molecules (74 upregulated, 90 downregulated) in the dexmedetomidine group. Among them, circ-Shank3 participated in the process of dexmedetomidine and improved POCD by regulating the P53 and NF-κB signaling pathways (Cao et al., 2020).

Overall, these results confirm the contribution of ncRNAs to dexmedetomidine-mediated neuroprotection in POCD, which could be potentially used for therapeutic targets (Table 3).

**TABLE 3:** Summary of ncRNAs associated with dexmedetomidine in postoperative cognitive dysfunction.

ncRNAs	Model	Main findings	References
miR 124, 132, 134, and 155	A rat model of LPS-induced neuroinflammation	The LPS-mediated increased expressions of miR 124, 132, 134, and 155 were significantly decreased after DEX treatment in both brain regions	Paeschke et al., 2017
miR-340	LPS-stimulated BV2 cells	Dex might exert anti-inflammatory effects in LPS-stimulated BV2 cells via upregulation of miR-340	Bao et al., 2019
miR-381	C57BL/6 J aged mice model with POCD	DEX may have a potential neuroprotective efect against POCD via the miR-381/EGR1/p53 signaling, shedding light on the mechanisms involved in neuroprotection in POCD	Wang et al., 2021b
miR-129	Rat model with POCD	Dex ameliorated SEV-induced POCD by elevating miR-129 and inhibiting TLR4 and NF-κB p65 phosphorylation	Wei et al., 2021c
miR-330-3p	-	Dex regulated cell apoptosis and mitophagy in Sev-induced neurotoxicity through the miR-330-3p/ULK1 axis	Xu et al., 2021
A total of 60 DE lncRNAs	Rat model with POCD	Dex alleviated POCD in rats and regulated lncRNAs expression profile in the hippocampus tissues of rats with POCD	Deng et al., 2020
164 DE circRNAs	Rat model with POCD	Circ-Shank3 participate in the process of Dex improved POCD through regulating the P53 and NF-κB signaling pathways and may potentially facilitate POCD treatment through the development of clinical drugs	Cao et al., 2020

circRNAs, circular RNAs; DE, differentially expressed; DEX, dexmedetomidine; LPS, lipopolysaccharide; lncRNAs, long non-coding RNAs; POCD, postoperative cognitive dysfunction; miRNA, microRNA.

## Discussion

POCD significantly affects the quality of life of patients, specifically, in elderly populations who have low tolerance for surgical stress. ncRNAs is involved in various pathological processes of POCD and can regulate neuroinflammation, neuronal apoptosis, oxidative stress, Aβ accumulation, and tau hyperphosphorylation through multiple signaling pathways.

However, there is a lack of direct experimental evidence on the role of ncRNAs in the BBB disruption during POCD. In addition, the impact of SARS-CoV-2 infection (COVID-19) on the CNS has been investigated because of the development of persisting symptoms in afflicted patients. RNA sequencing revealed that SARS-CoV-2 infection can alter ncRNAs expression profile in patients with dementia (Garofalo et al., 2021). The impact of the SARS-CoV-2 virus on neuroinflammatory responses related to cognitive disorders is associated with miRNA networks (Blount et al., 2022). However, the scientific evidence for the effect of ncRNAs on POCD patients with SARS-CoV-2 virus is still limited, though an association between SARS-CoV-2 infection and POCD has been revealed (Wei et al., 2021b). Further studies are required to address these gaps.

Prevention is the best method to avoid POCD. Non-harmful anesthetics and anesthetics with protective effect on cognitive function are critical for the prevention of POCD. However, evidence for the efficacy of anesthetics for POCD in humans is conflicting. For instance, although the use of dexmedetomidine (Zhao et al., 2020) and propofol (Tang et al., 2019) can decrease the incidence of POCD after major surgery without increasing any adverse effects, conflicting results have been observed in other clinical studies (Deiner et al., 2017; Sun et al., 2019). Noteworthily, anesthetics efficacy for POCD might depend on differences in ncRNAs expression patterns due to individual differences and type of anesthetic (see Brief Summary of the Role of ncRNAS in POCD**)**. Thus, to understand the underlying mechanisms it is necessary to provide strategies for the prevention and treatment of POCD.

Furthermore, exosomes are extracellular vesicles that can contain proteins, lipids, mRNAs and ncRNAs; these vesicles can cross the BBB and deliver therapeutic agents (such as ncRNAs) to treat neurological disorders (Qian et al., 2022). Recently, the therapeutic effect of exosomes on several neurocognitive disorders including Alzheimer’s disease (Yang et al., 2022), traumatic brain injury (Moss et al., 2021), and Parkinson disease (Chen et al., 2020) has been confirmed in pre-clinical and/or clinical trials. Yang et al. (2020) demonstrated that exosomes of antler mesenchymal stem cells can improve POCD in rats with cardiopulmonary bypass by inhibiting the TLR2/TLR4 signaling pathway. However, no further experiment has been performed to reveal their clinical effects for the treatment of POCD. In addition, evidence for the efficacy of other types of exosomes in POCD is limited. Thus, given the advantages of exosomes, comprehensive exploration into their efficacy on POCD should take priority.

In conclusion, ncRNAs are involved in regulating the expression of genes and markers that are critical in neurogenesis and undoubtedly play important roles in repairing neuronal damage induced by surgery/anesthetics. Mechanistically, they influence the development and progression of POCD through multiple signaling mechanisms in various cell types in the brain, although there is no doubt that more studies are warranted to obtain a more complete mechanistic picture. This study provides a summary of the evidence on the association between ncRNAs and molecular events observed in POCD. Treatments affecting ncRNAs may be useful in the management of POCD. Further research is required to develop a specific and non-toxic ncRNA strand that can be used in the clinical setting.

## Author contributions

Y-SY and S-LH prepared the manuscript and contributed to writing original—draft and visualization. W-CC, C-MW, and Q-MH drew the figures and tables and contributed to writing—review and editing. SL, Y-CS, and H-FH reviewed and finalized the manuscript and contributed to funding acquisition validation, and resources. All authors had full access to all the data in this review and took responsibility for the integrity of the data and accuracy of the data analysis.
